# INSERTION OF THE FATHER IN THE CARE OF THE HOSPITALIZED PRETERM INFANT: PERCEPTION OF THE MULTIPROFESSIONAL TEAM

**DOI:** 10.1590/1984-0462/;2019;37;3;00014

**Published:** 2019-06-19

**Authors:** Natalia Cristine Soares, Maria Piassa Lourenço Bernardino, Adriana Valongo Zani

**Affiliations:** a Universidade Estadual de Londrina, Londrina, PR, Brazil.

**Keywords:** Fathers, Patient care team, Infant, premature, Father-child relations, Pai, Equipe de assistência ao paciente, Recém-nascido prematuro, Relações pai-filho

## Abstract

**Objective:**

To understand the perception of the multiprofessional health care team regarding the inclusion of fathers in the care of preterm infants who are in Intensive Care Units (ICUs).

**Methods:**

This is a descriptive study with a qualitative approach, using a semi-structured interview with 12 health care professionals of a neonatal ICU, from February to July 2017. The data were analyzed according to the Discourse of the Collective Subject.

**Results:**

Seven main ideas (MI) emerged from the text analysis, which were grouped into two themes: 1) the role of the father according to the multiprofessional health care team views (MI1: parent provider, MI2: shared care, MI3: supportive father); 2) perception of the father caring for the hospitalized preterm infant (MI4: father does not change diapers; MI5: father conquering new spaces; MI6: strengthening the bonding; MI7: father providing maternal security.

**Conclusions:**

The results of this study point out to the importance of including the father figure in the humanized care of preterm infants. Professional health care team should be more aware of fathers’ importance in the care of hospitalized preterm infants.

## RESUMO

**Objetivo:**

Compreender a percepção da equipe multiprofissional referente à inserção do pai no cuidado ao filho prematuro hospitalizado na Unidade de Terapia Intensiva (UTI).

**Métodos:**

Estudo descritivo de abordagem qualitativa, por meio de entrevista semiestruturada feita com 12 profissionais que atuam na UTI neonatal, realizado no período de fevereiro a julho de 2017. Os dados foram trabalhados de acordo com o Discurso do Sujeito Coletivo.

**Resultados:**

Emergiram sete ideias centrais (IC), que foram agrupadas em dois temas: 1) o papel do pai na visão da equipe multiprofissional (IC1: pai provedor; IC2: o cuidado compartilhado; IC3: pai apoiador); 2) percebendo o pai no cuidado do prematuro hospitalizado (IC4: pai não troca fraldas; IC5: o pai conquistando novos espaços; IC6: fortalecendo o vínculo; IC7: o pai proporcionando segurança à mãe).

**Conclusões:**

Os resultados deste estudo apontam para a importância da inserção da figura paterna como proposta de assistência humanizada, estando os profissionais mais conscientes da importância do pai no cuidado do filho prematuro hospitalizado.

## INTRODUCTION

For a long time, the care of the child was sole and exclusive responsibility of the mother, while the father was responsible for supporting the family only. This stems from the culture of distribution of family roles, in which the father must be responsible for providing the family with all necessary support, and the mother for the raising and care of children.^1^

It is important to point out that the experience of fatherhood for men is felt and lived in a very particular way, that is, there is no single-father model; there are differences of perception depending on the country, social class and the age of parents.^2^ ^,^ ^3^ Fatherhood is an experience built on several levels, in which the sociocultural aspects are associated with being a provider of resources, respect and authority, and the bonding aspects are linked to the relationship with the mother. When a man becomes a father, there is a great change in their life, and it can occur from the moment of the news of pregnancy of their partner, through the birth of the child.^2^ This period is marked by the aggregation of new roles, which narrow the bond father-son. That being said, there is a social demand for contemporary fathers to exercise a more implied and active fatherhood when it comes to the coexistence with and the care of their infant.^1^ ^,^ ^2^

During pregnancy, parents dream about the time when they will meet their child, expecting them to be born strong and healthy; but the unexpected can happen, including the coming of a premature baby, who needs thorough and specialized care and hospitalization, which can lead to risks.^3^ This new situation can lead to feelings of fear, uncertainty and insecurity about the health of the newborn. The premature child is perceived by their father as fragile, small and immature.^4^ Faced with this, the father is afraid of touching his child and of losing them, but he wishes to be together. Male parents have confidence in the technological environment of the Neonatal Intensive Care Unit (NICU) and health professionals to maintain their child’s life. At the same time, the whole technological apparatus generates a separation from the child, who often is not allowed to be carried, which reduces physical contact with parents.^4^

The greater participation and consequent insertion of the father in the care of infants were not tracked by health professionals and the society itself at the same speed. The difficulties of professionals to include the father in the care of an infant within the hospital environment persist nowadays, and in the case of a premature newborn, it becomes more complex.^3^

The premature birth of a child in need of intensive treatment is a moment of tension for the whole family. Therefore, the exercise of motherhood and fatherhood, as well as the process of its development, can be affected especially when it comes to the father-baby bond. Thus, the health care team must be prepared to deal with the feelings and emotions of the parents and also to offer support.^1^ Careful attention, guidance and caring for the family are still centered on the mother figure nowadays; the father has always been only a coadjuvant. Learning to work with the new cultural reality in which men take care of their homes and children together with women, who in turn have careers, is a must.^4^

Therefore, the objective of this study was to understand the perception of the multiprofessional team about the insertion of the father in the care of a premature child being assisted at a NICU.

Hence the need for a study that prioritizes this reality, the role of the father in the life and care of a premature child while showing ther peculiar feelings, as relevant and real as the mother’s, although sometimes underestimated and forgotten by health care professionals. It is worth remembering that the insertion of the father is contemplated in Ordinance No. 930, dated May 10, 2012, by the Ministry of Health, which describes the encouragement of participation and protagonism of both the mother and father in the care of a newborn as a guideline.^5^

## METHOD

This is a qualitative study integrated into a broad research project entitled “The figure of the father in the care of premature and low birth weight neonates hospitalized at a Neonatal Intensive Care Unit”, funded by the National Council for Scientific and Technological Development (CNPq), with the general objective of capturing and systematizing the father’s participation in the care of premature newborns.

First, a father-aimed protocol of care was developed, including parental training in 14 types of care of a hospitalized premature infant, and validated by professionals specialized in neonatology and with extensive experience in clinical care of premature newborns. After validation, this protocol was presented to the multiprofessional team working at the neonatology department of the study institution, which was trained for a period of six months. Subsequently, the protocol was implemented and has been put to use in the institution since 2013. This research was initiated after approval by the Research Ethics Committee of *Universidade Estadual de Londrina* (UEL), with presentation of the Certificate of Presentation for Ethical Assessment (CAAE) nº 30709814.0.0000.5231, according to opinion no. 694,303, and signing of the free and informed consent form by participants.

The study was carried out at the NICU of a tertiary university hospital in the northern region of Paraná. Accredited by the Brazilian Public Health System (SUS), this hospital acts by providing health care in practically all medical specialties, human resources training, continuing education, research and technological development, besides offering technical and scientific cooperation within the health services network of Londrina through services such as laboratory tests, research and treatment of genetic diseases. Its structure is medical-surgical and pediatric hospitalization units, maternity, surgical center, emergency room and adult, pediatric and neonatal ICUs. The neonatal unit has ten intensive care beds, ten intermediate care beds and four intermediate care “kangaroos”.

Participants of this study were professionals working at the NICU: neonatologists, resident physicians in the 2nd year of Neonatology, resident nurses of the 2nd year of Neonatal Nursing, psychologists, physical therapists, social workers and nursing technicians. These professionals were invited to integrate the study by the researchers and were informed about the objectives, data collection procedures, confidentiality of information, possible risks and possibility of interruption at any time, without prejudice to the care of parents and hospitalized infants. We included health professionals with minimum of one year of experience in clinical care of premature newborns at the NICU. Professionals who were absent by leave or vacations at the time of data collection were excluded from the study.

The average duration of researchers’ meetings with the participants was 30 minutes, considering the initial interaction and the interview itself. Data were collected from February to July 2017, through a semi-structured interview divided in two parts: the first one concerning the characterization of professionals; the second, addressing the objectives themselves. The main guiding questions of the interview to motivate the professionals’ speech were: what is your opinion about the insertion of the father/man in the care of a child being cared for at a NICU? How do you perceive the presence of the father at the NICU? For you, what is the role of the father at the NICU during a child’s hospitalization? What do you think the father can do at the NICU? Have you ever performed or witnessed any activity with a father regarding his involvement in their child’s care?

The interviews were recorded in a digital recorder and descried in a field notebook for researcher’s synthesis. At the end of the interview, the professional was asked to listen to the recording and to read the synthesis, being granted the right to change information if they deemed necessary. The interviews took place in locations chosen by the professionals (meeting rooms, offices and multidisciplinary rooms).

The data were analyzed according to the theoretical reference of Social Representations, which presents a strong linking with the objects of study in the field of health, since it can capture the most subjective aspects permeating the problems inherent to this area. Social Representations are a series of opinions, explanations and affirmations produced based on the daily routine of groups, being the communication the primordial element in this process. Considered as a common-sense theory, as it is created by groups as a way of explaining reality, the Social Representations validate a particular form of knowledge whose function is to create behaviors and communication between individuals.^6^

In order to learn and describe the perception of the multiprofessional team about the insertion of the father in the care of a premature newborn hospitalized under the Social Representations, the Collective Subject Discourse (CSD)^7^ was the method chosen to build meanings, allowing the approximation of the phenomenon under study. CSD proposes the organization and tabulation of qualitative data in a discursive way, attempting to clarify what one thinks or the experiences of a certain group on a given theme.^7^ For this study, three methodological figures were used: the key expression, the central idea, and the CSD. The key expression is the methodological figure that reveals the essence of the statement, that is, what the subject spoke about a particular topic. The central idea describes the meaning present in the key expression.

In CSD, the qualitative data are presented through a synthetic discourse, written in first person singular and elaborated with the most significant extracts of statements of similar meaning. It is based on the theory of Social Representations and analyzes the central ideas, anchorages and similar key expressions present in individual discourses.^7^ ^,^ ^8^

To arrive at the synthesis, two instruments of discourse analysis were used. In the discourse analysis tool 1, the key expressions identified in each interview were transcribed, and they expressed the axes defined for analysis. After this transcription, the central ideas of each key expression were highlighted. In the discourse analysis tool 2, the key expressions of all interviews referring to the highlighted central ideas were grouped and transcribed literally, which allowed to formulate a CSD for each central idea. To formulate the CSD, the key expressions were put together aiming to form a coherent discourse, so connectors were used to provide the CSD with meaning without altering the structure of sentences elaborated by the subjects.

## RESULTS

All health professionals of the unit were invited to participate in this study (n=38), four nurses, three residents of the second year of neonatal nursing, six neonatologists, three residents of the second year of neonatal medicine, 16 nursing technicians, one psychologist, one social worker, and four physical therapists. Of these professionals, two nurses were excluded because they had less than one year of experience in the service, one resident of neonatal nursing due to start of vacation period, five neonatologists (two were on vacation, two refused to participate, and one was in a scientific event), a resident of Neonatal Medicine who was currently carrying out activities in another unit, three physical therapists (two for being in service for less than one year, and one for being on paid leave), and 14 nursing technicians, since two were on vacation, four refused participation, two were on medical leave and six said they were unavailable because of the activities in the unit. Therefore, the sample was formed by 12 professionals.

As for the characteristics of the 12 professionals, there was predominance of females (91.6%). Mean age was 34.2 years (ranging from 22 to 51), and the mean number of years of work in the NICU was 6.8 years (ranging from 1 to 24). As for schooling, all professionals, including technicians, had higher education. Only professionals in technical level did not have a postgraduate degree. General information of the participants is listed on Table 1.

**Table 1:** Characteristics of the multiprofessional team.

	Description	n (%)
Gender	Female	11 (91.6)
Professional category	Neonatologist nurse	2 (16.7)
	Resident of Neonatal Nursing (2nd year)	2 (16.7)
	Neonatologist physician	1 (8.3)
	Resident of Neonatologist Medicine (2nd year)	2 (16.7)
	Social worker	1 (8.3)
	Psychologist	1 (8.3)
	Physical therapist	1 (8.3)
	Nursing Technician	2 (16.7)
Marital status	Married	3 (25)
	Single	9 (75)
Children	None	9 (75)
	1	2 (16.6)
	2	1 (8.4)
Family income (in minimum wages)	3-5	7 (58.3)
	5-10	2 (16.6)
	+10	3 (25.1)
Age group (years)	20-30	5 (41.7)
	31-40	3 (25)
	41-50	3 (25)
	51-60	1 (8.3)
Background in neonatal ICU (years)	1-5	8 (66.4)
	6-10	1 (8.4)
	11-15	1 (8.4)
	16-20	1 (8.4)
	21-25	1 (8.4)
Schooling	Higher education	12 (100)
Postgraduate studies*	*Lato sensu* (Specialization concluded)	3 (20)
	*Lato sensu* (medical residency concluded)	4 (26.7)
	*Lato sensu* (ongoing medical residency)	4 (26.7)
	*Stricto sensu* (ongoing Master’s)	1 (6.7)
	*Stricto sensu* (Master’s concluded)	1 (6.7)
	None	2 (13.2)

ICU: Intensive Care Unit; *Some professionals have more than one postgraduate modality degree.

At the end of data collection and analysis process, CSD had collective testimonies based on individual statements, conveying an opinion or a positioning, with the purpose of producing the effect of collective opinion in the message receiver, as if spoken directly by a single subject of speech. Therefore, seven central ideas emerged from the discourses analyzed, which were grouped into two themes:

- The role of the father in the view of the multiprofessional team (CI1: the provider, CI2: shared care, CI3: supportive parent).
- Perceiving the father in the care of hospitalized premature infants (CI4: father does not change diapers; CI5: the father conquering new spaces; CI6: strengthening the bond; CI7: the father providing the mother with security/confidence.

To better understand the analysis conducted and preserve the anonymity of participants, the their names were replaced by the first letter representing their category-resident of Neonatology (RN), psychologist (P), social worker (SW), nurse technician (NT), neonatal nurse (NN), neonatologist (NM), resident of Neonatal Nursing (RNN) and physical therapist (PT)-followed by a numerical sequence when more than one professional belonged to the same category, as per the order of interviews.

### Theme 1: The role of the father in the view of the multiprofessional team

####  CI1: the provider 

####  CSD1

The father should be focused on work, in order to leave the mother totally free for the care of the child, focusing on the financial side, since he should be the breadwinner of the family (RN1, NT1).

One can see in CSD1 that, for some professionals, the role of the father has not changed over the years, since they believe he should be the provider of support and should not be included in the direct care of the child, thus delegating care of the baby exclusively to the mother. However, a significant number of professionals have shown the opposite perception, saying that the father now has a broader role, which is not just the provision of financial resources. According to CSD2, the father should share the care, for he is a member of the family with the same importance as the maternal figure.

####  CI2: shared care 

####  CSD2

The role of the father is as fundamental as the role of the mother. Nowadays, the father must to be increasingly inserted in the care of babies, mainly due to the social reality in which, more and more, the husband/father has played the role of helping the woman in the home. The woman, nowadays, has other professional activities, beyond caring for the child. So fathers are increasingly aware of the baby’s care so they can help their wives at that moment. There are fathers in the NICU who take care of their children by themselves, due to the death of their wife (PT, SW).

In DSC2, the professionals share the opinion that the father plays a fundamental role in the birth and hospitalization of the premature child. The father is considered to be supportive and thus shelters his wife/companion and the rest of the family.

####  CI3: the supportive father 

####  CSD3

Fathers face the challenge and help. The support they provide is extremely important. The participation of the father in this initial phase, mainly in relation to the NICU, in the sense of being able to support and shelter the mother, share this difficult moment with her, full of emotions. The figure of the father is as important as the mother. The father is often on the side of the mother to support and learn how to care for the baby even when the baby is seriously ill. The father is as important as the mother, especially in the first contacts, because the mother is already a little fragile in postpartum, sometimes in post-surgical recovery, so the father is the one who makes this bridge for the family, especially for the mother in the first days. The presence of the father is fundamental because the mother usually finds herself in a difficult time. In addition to seeing her baby hospitalized, the moment she leaves, she hopes, like every mother, to take the baby with her. And in the case of a premature, this does not happen. She has to leave the little baby, go home and live her life. So the father by her side, supporting her, giving strength, having positive thoughts and helping her in the day to day is essential. In addition, the father who experiences the care in the hospital gives much more support at home (SW, NM, NT2, NN1, NN2, RN2).

When the father is inserted in the environment of a neonatal unit, he experiences a mix of emotions and challenges just like the mother, and, faced with this new situation, professionals perceive the presence of the father in the care and its evolution in this new process.

### Theme 2: perceiving the father in the care of hospitalized prematures

####  CI4: The father does not change diapers 

####  CSD4

At first, some fathers do not want to be inserted, and one of the causing factors is cultural. They think that certain types of care should not be performed by men. Some parents even mention: imagine me changing diapers, I will not do that! I have to work to support the Family, not change diapers, this is the mother’s duty (NN, NM, RNN1).

Not only professionals have pre-established conceptions based on their familial, professional and social experiences. Some fathers refuse to participate directly in the care of the child, claiming that, culturally, the father should be the provider of the family. Changing diapers should not be performed by them, as noted in the previous speech. In contrast, social and cultural changes have been occurring. Consequently, the father has gained new spaces, as stated in CSD5, also gaining the benefit of the extended paternity leave in order to accompany their child in the neonatal unit as a result of the loss of their wife. This achievement was considered by the professionals as positive, as it enabled the father to follow the entire process of development of their child in the neonatal unit.

####  >CI5: the father conquering new spaces 

####  CSD5

Through a referral, a father’s paternity leave was obtained by the [National Social Security Institute] INSS for four months so that he would stay with the baby for four months due to the death of his wife, this being one of the first cases we achieved and with wonderful result for the baby. The emotion of the father when he managed to bathe his baby was incredible! The presence of the father is a must for the development of the personality of the child. I believe that the father must participate in all activities, except breastfeeding itself. Obviously the father can not do that, but he can be inserted into the whole breastfeeding process to help his wife while the baby is being breastfed (SW, NT1, NM, PT).

Some fathers find it more difficult to feel like a parent and only begin to experience paternity after the birth of the child, unlike mothers, who, in general, begin to feel in the rola of a mother from the moment they discover gestation. The professionals report that performing small care tasks with the premature child in the hospital environment strengthens the bond father-child, and they begin to live fatherhood effectively, as represented in CSD6.

####  CI6: strengthening the bond 

####  CSD6

In caring for the baby, the father feels that he has a child and that he needs to develop a bond. When the father is effectively inserted in the care, the bond becomes much stronger. And not only the bond with the child, but also with his wife. He can bathe the baby, administer medications that the baby will take at home, learning all before the discharge of the baby. Fathers are inserted from the time the baby starts to get more stable. It is important that they are in touch, so they can change diapers, clean their eyes, participate in the bath time, help the mother in while the baby can not suck, kangaroo, caress, things that strengthen the bond (NN1, NN2, RNN1, NM, RNN2, RN2, PT, SW, P).

Some health professionals see the presence of the father as a source of security/confidence for the mother, who, at the time of birth and hospitalization of the child, is fragile. They say that the father, in general, is more rational and helps the mother to understand this new situation, as pointed out in CSD7.

####  CI7: the father sheltering the mother 

####  CSD7

The participation of the father within the NICU is fundamental, mainly to give security/confidence to the mother. Due to the fact that the father is considered to be the side of reason, he is able to better assimilate information about the severity or improvement of the child and pass it on to mother in a way that makes her feel safe, because she is receiving this information from the father (NT1, NT2, NN1, NN2, P, NM, RN1, RN2, PT).

## DISCUSSION

The results show divergences of opinion among the multiprofessional team members regarding the role of the father in NICU. Some professionals believe that the father should play the role of provider only and, therefore, there is no need to insert them in the care of children. In contrast, other professionals recognize the importance of the role of the father and believe that this care must be shared. A study^9^ on the social representations of fatherhood built by health professionals reinforced the results of this investigation when addressing the professionals who see the “new” father, who does not want to be only the provider of the family, and wishes to participate in all stages of life of their children, helping in the care. This father wants to be present from the moment of gestation, at birth and in the first years of life of his children, however society and even health professionals, in face of this fact, have been resistant to this new vision, not valuing the presence of the father in this new context.

The presence of the father has contributed positively to the child’s birth, assisting with protective responses, fostering intimacy with both the newborn and improving their marital relationship, strengthening bonds.^9^ ^,^ ^10^ Sharing tasks with the mother, by educating and caring for the child, shows both society and many professionals the real importance of having fathers present in the life of a newborn, from a simple diaper change to the integral care of the child regardless of the environment, helping their companions, who often are taken by an emotional and physical overload.

The participants of this study also affirm that the father plays the role of supporter of the mother and the family in this conflicting and unique moment. The father’s presence during hospitalization is important not only as an emotional support for the mothers, but also to promote the fatherly bond, knowledge and safety in the care that will be provided after hospital discharge^11^.

Concerning professionals’ perception about the care of hospitalized premature infants, the speech of some of them attest that in some situations, due to cultural prejudices, at first some fathers do not wish to participate in the care of the child, since they believe they should be financial providers and the mothers should be the caregivers. However, health professionals have noticed that many fathers are conquering new spaces, as represented in one of the speeches about a father who lost his wife and obtained extended paternity leave so that he could assist in the care of his hospitalized premature infant.

The difficulty of having the father released from work activities is an obstacle to the bonding with his premature child, his companion and permanence with them. In Brazil, paternity leave is guaranteed by the Federal Constitution of 1988, article 7, granting the father a period of five working days after childbirth. However, this period is insufficient to meet the needs demanded by the mother and the child in the first moments after birth, and this is even more delicate when it comes to premature birth.^12^ ^,^ ^13^ In the family configuration, a new paternal figure has emerged, expressing more affection and participation in the care of the family, contrary to the hegemonic paternity model, which used to consider man as a financial provider, distancing them from fatherhood and from the responsibility in the development of children.^14^

The multiprofessional team participating in the study, in general, has realized that the insertion of the father leads to strengthening bonds not only with the child, but also with the mother, an important fact, since these parents experience the news of a premature birth and the need of hospitalization of the child in the NICU as a moment of surprise and great concern.^15^ In seeking to establish links between father and son, the multiprofessional are able to make the environment more conducive to the contact between them, thus helping to create a bond. This occurs mainly through the stimulation of father figure, by inserting them into simple care tasks, such as changing diapers, hygiene of the baby’s eyes, and aiding in the breastfeeding process.^16^ ^,^ ^17^

This study showed that the multiprofessional team perceives the father as a source of safety/confidence for the mother, being a factor that can minimize maternal stress. Another study that described fathers coping with children admitted to a neonatal unit in Norway identified that mothers who had their partners at their side during hospitalization of the premature child showed reduction of stress and more confidence/safety about the restoration of premature infants’ health.^18^

As strength of the study, the multiprofessional team was shown to be mostly favorable to the insertion of the father in the care of the child during hospitalization in the NICU, understanding that this brings benefits for the infant and for mother by strengthening bonds.

Among the limitations of the study, the difficulty in conducting interviews due to work overload and reduced number of professionals in the scenario studied is highlighted. However, this research allowed to identify that professionals understand the importance of the presence and participation of the father in the neonatal unit, which may help other researchers and professionals of the institution, as well as other services, to turn their attention to this topic.

With the results herein presented, one may conclude that the objective of the study was achieved by obtaining, through Social Representations, the perception of the multiprofessional team regarding the insertion of the father in the care of the premature child in the NICU. The multiprofessional team still has some prejudices about the role of the father in the family context, understanding that they should be the financial provider, but most team members understand that the roles of the father/man went through major changes. Nowadays, the father wants to participate in the care of the premature child, as well as support his companion in this unique and complex moment. This more effective participation is beneficial for the premature baby and the whole family, as well as for the relation between the health team and the family, as there is more understanding of information about the clinical evolution of the child.
